# Microencapsulated Healthy Oil Mixtures to Enhance the Quality of Foal Pâtés

**DOI:** 10.3390/foods11213342

**Published:** 2022-10-24

**Authors:** Aurora Cittadini, Paulo E. S. Munekata, Mirian Pateiro, María V. Sarriés, Rubén Domínguez, José M. Lorenzo

**Affiliations:** 1 Instituto de Innovación y Sostenibilidad en la Cadena Agroalimentaria (IS-FOOD), Universidad Pública de Navarra (UPNA), Campus de Arrosadia, 31006 Pamplona, Spain; 2 Centro Tecnológico de la Carne de Galicia, Avd. Galicia No. 4, Parque Tecnológico de Galicia, San Cibrao das Viñas, 32900 Ourense, Spain; 3 Área de Tecnoloxía dos Alimentos, Facultade de Ciencias, Universidade de Vigo, 32004 Ourense, Spain

**Keywords:** foal meat product, healthy pâté, physicochemical properties, nutritional value, sensory acceptance

## Abstract

This study aimed to evaluate the use of microencapsulated oil mixtures as partial animal fat replacers and their effects on the physicochemical, nutritional and sensory qualities of foal pâtés. Three different batches were manufactured: a control (CON) formulation, with foal dorsal subcutaneous fat (30 g/100 g), and treatments 1 and 2 (T1 and T2), with 50% of the animal fat replaced by microcapsules containing algal oil mixed with walnut oil (T1) or pistachio oil (T2). The reformulated samples presented significant (*p* < 0.001) diminutions of fat contents, which achieved reductions of 34.22% (“reduced fat content”) and 28.17% in the T1 and T2 samples, respectively, and the lipid reformulation did not affect (*p* > 0.05) the texture or lipid oxidation of the samples. Furthermore, both microencapsulated oil mixtures significantly (*p* < 0.001) reduced (11–15%) saturated fatty acid (SFA) concentrations and increased (*p* < 0.001) mono- (T2) and polyunsaturated (T1) fatty acid contents (8% and 68%, respectively), contributing to the obtainment of nutritional indices in line with health recommendations. Additionally, consumer acceptability did not display significant (*p* > 0.05) differences among samples. Hence, the outcomes indicated that the incorporation of these microencapsulated oil mixtures as partial animal fat replacers, especially the T1 mixture, represents a promising strategy to obtain healthier foal pâtés, without compromising consumer approval.

## 1. Introduction

Today, the food industry, and in particular the meat sector, have to satisfy ever more demanding consumers. On the one hand, there is a high demand for tasty and attractive ready-to-eat foods, due to the fast-paced modern lifestyle [1,2], while at the same time people are more interested in the quality, nutritional value and environmental footprint of the food they consume [3].

In this context, pâté represents a very popular ready-to-eat meat product and is consumed worldwide owing to its affordable price and its appreciated sensory properties, such as its intense flavor and smooth texture [4]. However, this product, although it could represent an important source of protein, vitamins A and B, and iron, contains high amounts of fat (25–45%), principally saturated fatty acids (SFAs—above 30%), cholesterol and other components which, if habitually consumed, could promote adverse health effects, such as the onset of obesity, cardiovascular illnesses and other chronic diseases [5,6].

Therefore, in order to improve the nutritional value of this type of product, the meat industry and the scientific community are focusing on innovative strategies to achieve meat products with added value but without compromising textural and sensory features [5]. In this regard, lipid reformulation represents one of the main tactics employed with the purpose of improving lipid fractions both quantitatively and qualitatively. In fact, this type of reformulation normally entails the diminution of animal fat contents and their replacement with other fat sources in line with health recommendations, such as vegetable and/or marine oils, which have been demonstrated to produce a significant reduction in both fat and SFA contents (improving fatty acid profiles) in meat products [1]. Nevertheless, animal fat, a solid fat source, plays a key role in these products, not only from a technological standpoint but also from a sensorial perspective, since it is a basic ingredient that significantly affects attributes such as texture, taste and flavor of meat products [7,8]. Hence, replacing animal fat with other fat sources represents a considerable technical challenge. For this reason, a partial replacement is generally carried out in the case of pâté, since its total replacement with oils can produce adverse sensory quality effects [9]. Furthermore, it is well-known that vegetable and marine oils are characterized by high levels of healthy but also unsaturated fatty acids (i.e., monounsaturated—MUFA and polyunsaturated—PUFA), and are, for this reason, naturally prone to oxidation, instability and degradation [10]. As a consequence, the technological features, oxidative stability and sensory qualities of pâtés could be impaired by this lipid reformulation [11]. In this context, encapsulation has recently stood out as a promising tool for oil incorporation, since it is able to overcome the above-mentioned problems [12]. Moreover, this technique could be considered suitable for the incorporation of oils in this type of product, since pâté is an emulsified meat product and does not require a fat replacer that mimics the animal fat aspect [1]. In addition, recent publications [1,13] have highlighted that the use of oil combinations, instead of pure and singular oils, can favor the enhancement of the nutritional profiles of final products as well as moderate their potential impacts at technological and sensorial levels. Hence, microencapsulated oil mixtures are proposed as potential partial animal fat replacers in foal pâtés. The selection of oils is another pivotal step in lipid reformulation, and, for this work, algal oil was blended with walnut or pistachio oils. Algal oil is a well-known and precious source of long-chain omega-3 fatty acids (LC *n*-3), such as eicosapentaenoic (EPA—C20:5*n*-3) and docosahexaenoic (DHA—C22:6*n*-3) acids [14,15,16], which are recognized for their beneficial health effects, including reducing the risk of developing chronic diseases, such as arthritis and cardiovascular problems, and improving brain function [17]. Walnut and pistachio oils represent “gourmet” and in vogue novel healthy oils. In particular, walnut oil is characterized by remarkably high contents of PUFAs, mostly the essential linoleic acid (C18:2*n*-6) fatty acids, whilst, in pistachio oil, MUFAs represent the most plentiful portion and oleic acid (C18:1*n*-9) is the predominant fatty acid. These oils are also related to beneficial effects on human health, such as reducing the incidence of coronary heart disease, hypertension, metabolic syndrome, cancer and other inflammatory conditions [18].

Taking into consideration the purpose of reducing the fat contents and improving the lipid profiles of pâtés, the use of the meat, liver and fat from an animal species characterized by a valuable nutritional profile, such as foal, could represent another positive step towards the achievement of a healthier final product. Foal meat, in fact, is recognized for its low fat and cholesterol levels, among other attributes [19]. Furthermore, equids stand out for their unique digestive physiology which favors the efficient absorption and transfer of dietary *n*-3 PUFAs from feed (pasture) to muscle tissues, with limited deposition of *trans*-fatty acids [20]. Finally, the use of these animals also meets the environmental concerns of consumers, since they are considered an eco-friendly species [3,20]. In this sense, foals could be considered a “sustainable” source of high-quality ingredients with a favorable lipid profile for the preparation of healthy pâtés.

In addition, hitherto, there has been a shortage of studies on the application of vegetable and/or marine oils in foal meat products [15] and there have been no studies at all on foal pâtés. This is also the first time that these fat sources (both foal dorsal fat and the selected oils) have been employed in this type of meat product, and there have been no reports on the formulation of healthy foal pâtés using these encapsulated oil mixtures as partial animal fat replacers.

Therefore, the purpose of this work was to assess the effects of partial replacement of animal fat by microencapsulated oil mixtures on the proximate composition, physicochemical properties, nutritional profiles and sensory acceptability of foal pâtés. At the same time, this investigation had the intention of valorizing the equid species and foal meat as well as its other “components” (liver and dorsal fat) and derived processed products.

## 2. Materials and Methods

### 2.1. Microencapsulation of Oil Mixtures

For the present study, two oil mixtures were microencapsulated, walnut (treatment 1—T1) and pistachio oils (treatment 2—T2) being blended with algal oil. The walnut oil (Naturgreen, Librilla, Murcia, Spain) and pistachio oil (Tenuta del Roero, F.lli Ruata S.p.a, Beldissero d’Alba, Cuneo, Italy) were bought at a local market, while the algal oil, recognized for its high *n*-3 concentrations (418.3 mg DHA/g oil), was kindly provided by the Solutex Corporation (Madrid, Spain). The oil microencapsulation was performed following the method proposed by Vargas-Ramella et al. [21]. Firstly, 50 g of sodium caseinate and 50 g of lactose were dissolved in 400 g of distilled water using an Ultra-Turrax^®^ T 25 digital (IKA^®^-Werke GmbH & CO. KG, Staufen, Germany) at 12,000 rpm during 10 min. Successively, 50 g of oil mixture (46.5 g of walnut oil—T1—or pistachio oil—T2—blended with 3.5 g of algal oil) and 250 g of distilled water were gradually added, with the mixture maintained in continuous agitation until a homogeneous and stable emulsion was obtained. Finally, the emulsions (in constant agitation) were dried using a Mini Spray-dryer, Mod. B-290 (Buchi AG, Flawil, Switzerland) in order to obtain the microencapsulated oil powders. The feed rate was set at 30% (9 mL/min) and the inlet temperature at 145 °C and the aspirator was adjusted to 80% (32 m^3^/h). In addition, the microencapsulation yield was on average 95.74%. The dried powders obtained were vacuum-packed (V900, Tecselor S.L., Murcia, Spain) until pâté manufacture.

### 2.2. Manufacture of the Foal Pâtés

**Figure 1:**
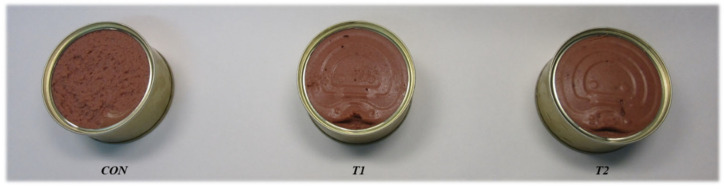
Appearance of foal pâtés. CON: pâté prepared with 100% foal dorsal fat; T1: pâté prepared with 50% of foal fat replaced with microencapsulated walnut and algal oil mixture; T2: pâté prepared with 50% of foal fat replaced with microencapsulated pistachio and algal oil mixture.

Three different treatments of pâté were manufactured in the pilot plant of the Centro Tecnolóxico da Carne (CTC) (Ourense, Spain) (Figure 1): the control (CON) was formulated with foal dorsal subcutaneous fat (30 g/100 g), while in treatments 1 and 2 (T1 and T2) 50% of the animal fat (15 g/100 g) was substituted with the corresponding microencapsulated oil mixtures (15 g/100 g), consisting of algal oil (7% of the mixture) blended with walnut oil (T1) or pistachio oil (T2). The whole experiment was repeated with the same ingredients and formulations in four batches in distinct months (3 treatments × 9 repetitions × 4 runs).

The chemical composition and lipid oxidation of the fat sources employed (Table 1) and the fatty acid profiles of the foal fat and oils included in the manufacturing process (Table 2) were determined according to the same procedures used for the pâté samples (Section 2.3 and Section 2.4, respectively). All batches were elaborated with the same ingredients, apart from the fat source, as mentioned above. In detail, the foal pâté formulation consisted of lean meat (20 g/100 g), a fat source (foal fat and/or microencapsulated oil mixtures, depending on the batch) (30 g/100 g), foal liver (33 g/100 g), tap water (11.46 g/100 g), NaCl (2 g/100 g), milk powder (2 g/100 g), sodium caseinate (1 g/100 g), sodium phosphate (0.50 g/100 g), sodium nitrite (0.015 g/100 g) and sodium ascorbate (0.025 g/100 g). The lean meat, subcutaneous fat and liver were from Burguete foals provided by Cárnicas Mutiloa (Rocaforte, Navarre, Spain). 

All treatments were manufactured following the procedure described by Domínguez et al. [22], with some modifications. Briefly, the foal fat, lean meat and liver were chopped into small cubes, vacuum-packed separately and pre-cooked at 80 °C in a hot water bath for 30 min (fat during 10 min). At this point, the liver, the lean meat and the water (obtained from the pre-cooking) were added in a cutter (Sirman, mod C15VV, Marsango, Italy) and homogenized. Then, NaCl, sodium nitrite and sodium ascorbate were included, and a pre-emulsion was carried out, subsequent to which the rest of the ingredients—sodium caseinate, milk powder and potassium phosphate, foal fat and/or microencapsulated oil mixtures and tap water (at 65 °C)—were gradually added and chopped until a homogeneous batter was obtained. At this point, the pâtés were manually distributed into metal cans which were completely filled (100 g) and hermetically closed before thermal treatment at 80 °C for 30 min in an autoclave (Ster PE 50–100 mini, ILPRA, Barcelona, Spain). After the heat treatment, the samples were cooled in a blast chiller (IS101L-ONRO, Angelo Po–SAGI Spa, Ascoli Piceno, Italy) (−21 °C/30 min) and stored at 4 °C until they were analyzed (the day after). Nine cans of pâté for each treatment and each elaboration run were included in the analysis. 

### 2.3. Proximate Composition, Physicochemical and Lipid Oxidation Analyses

Moisture [23], protein [24] and ash [25] contents were assessed according to the International Standards Organization (ISO), while total fat was extracted and quantified following the approved procedure Am 5-04, established by the American Oil Chemistry Society [26]. The methodology reported by Domínguez [27] was followed to measure color and pH, which were determined at three different points for each sample. For the texture profile analysis, a penetration test was performed in line with the indications described by Vargas-Ramella et al. [21]. The test was carried out in triplicate. The rheological parameter procured from the corresponding force–penetration curves represented the penetration force to rupture (N). The protocol described by Vyncke [28] was applied to assess the thiobarbituric acid reactive substance (TBARS) values for the detection of the lipid oxidation rates of the samples, and results were expressed as mg MDA/kg sample.

### 2.4. Fatty Acid Analysis

As regards fatty acid determination, fat extraction and its transesterification were performed according to the procedure described by Domínguez et al. [29]. A gas chromatograph (GC-Agilent 7890B, Agilent Technologies, Santa Clara, CA, USA) equipped with a flame ionization detector (FID) and a PAL RTC-120 autosampler with a liquid injection tool (Pal System) was employed for the separation and quantification of fatty acids methyl esters (FAMEs) following the chromatographic conditions indicated by Domínguez et al. [29]. For FAMEs separation, a DB-23 fused silica capillary column (60 m, 0.25 mm i.d., 0.25 μm film thickness; Agilent Technologies) was used. The outcomes were expressed as g/100 g of fat. Furthermore, the nutritional indices *n*-6/*n*-3 and PUFA/SFA ratios, atherogenic (AI) and thrombogenic (TI) indices and hypocholesterolaemic/hypercholesterolaemic ratios (h/H) were determined as described by Cittadini et al. [15]. These indices are useful since they provide information about the action of some fatty acids which can prevent or promote coronary thrombosis and atherosclerosis, depending on their effects on low-density lipoprotein (LDL) concentrations and serum cholesterol. In particular, the impacts of fatty acids on cardiovascular risk are indicated by AI and TI indices, while h/H ratios reflect the functional effects of fatty acids on cholesterol metabolism [30,31,32].

### 2.5. Consumer Evaluation of the Foal Pâtés

The sensory evaluation was carried out with the purpose of evaluating consumer acceptance and preferences in relation to the different pâté samples. These studies were performed in the sensory laboratory of the Meat Technology Center of Galicia (Ourense, Spain) equipped with individual booths under white light, in line with the UNE-EN ISO 8589:2010/A1:2014 [33] standard. The sensory evaluation panel was composed of 60 consumers (29–45 years old from both genders) from Ourense (Spain). Unfortunately, the coronavirus global health emergency (June 2021) limited the presence of a greater number of participants. However, in accordance with Mammasse and Schlich [34], a suitable number of panelists was achieved. An acceptance test was performed in order to evaluate the reformulated foal pâtés, considering the following attributes: visual aspect, odor, taste, texture, greasy character and overall acceptability. For this analysis, the tasters used a 7-point hedonic scale, ranging from “1-disliked much” to “7-liked much”, according to Lago et al. [35]. In addition, for any above-mentioned descriptor, the panelists were asked to order the samples in relation to their preferences [36], employing a 3-point scale (1 = least favorite and 3 = favorite). The pâtés were served to each panelist in ceramic white dishes coded with 3-digit random numbers, using different sample distributions in order to avoid a “carry-over effect” [37]. Furthermore, unsalted bread and room temperature mineral water were presented/given to each assessor to rinse the palate and remove residual flavors at the beginning of the test and between samples.

### 2.6. Statistical Analysis

All statistical analysis were carried out employing the SPSS statistical software (SPSS 25.0, Chicago, IL, USA). Shapiro–Wilk and Levene tests were previously applied in order to verify normal distributions and variance homogeneity. The results were examined employing an analysis of variance (ANOVA) with a mixed model, where the parameters were introduced as dependent variables and the treatment was set as the fixed effect and replications as the random effect, while, for the acceptance test, consumers were considered as the random effect (each panelist tasted three samples, one for each treatment, in a single session). The pairwise differences between least squares means were assessed by Duncan’s method. Differences were considered significant if *p* < 0.05. In the case of the preference test, the data were subjected to the Friedman statistical test with Newell and MacFarlene tables (α = 0.05) in order to understand the disparities in terms of the panelists’ sensitivities regarding the three treatments of pâté. When a significant effect (*p* < 0.05) was detected, a least significant difference (LSD) test was applied as a multiple comparisons test.

## 3. Results and Discussion

### 3.1. Proximate Composition and Physicochemical Parameters of the Foal Pâtés

Table 3 shows the proximate composition and physicochemical results for the foal pâtés. As can be seen in Table 3, the employment of microencapsulated oils as animal fat partial replacers had a significant (*p* < 0.001) impact on the chemical composition of the foal pâtés.

A significant (*p* < 0.001) diminution in moisture values in the T1 and T2 samples was observed, with the highest percentages being reported for the CON batch in comparison with the reformulated samples. This trend could be justified by the fact that the microencapsulated oils (dried powders) showed extremely lower water contents in comparison with the animal fat (Table 1), as could be foreseeable. A similar trend was observed previously by other researchers who partially substituted pork fat for fresh fish oil [27] and for ethyl cellulose and beeswax oleogels (mixtures of olive, linseed and fish oils) [7]. Nevertheless, an opposite tendency was observed in pâtés where microencapsulated healthy oils (tiger nut, chia and linseed oils) [21] and a beeswax oleogel (linseed oil) [4] were employed as partial pork fat replacers. These discrepancies could be related to different factors, such as the manufacturing procedure, the different structures and compositions of oil-based animal fat replacers, as well as the different types of meat and animal fat employed. However, in the present study, the moisture contents (49.71–52.22%) were consistent with the outcomes reported by other authors for the same product, who recorded values of 50–53% [22,27,38].

Considering the fat content, a significant (*p* < 0.001) decrease in fat percentages was detected in the T1 and T2 batches, which achieved diminutions of 34.22% and 28.17% compared with the CON group, respectively. In this sense, T1 samples, for which the lowest fat amounts were recorded, can be claimed to have “reduced fat content” in line with Regulation (EC) No. 1924/2006 [39]. In this context, these outcomes represent a positive goal from a nutritional standpoint and the fulfilment of one of the objectives of this work. This reduction could be expected considering that the lipid content of the fat source employed in this study, namely, the horse fat, which had a total fat percentage of 85.56%, was partially substituted by microencapsulated oils composed of approximately only 21–23% of fat (Table 1). This tendency is, in fact, in line with the results previously observed by other authors [4,21,40,41], while the use of oils, such as olive or fish oils, directly in the meat mass of the product caused an increase in fat [22,27]. With this in mind, it is worth noting that the method selected for fat replacement could have a crucial effect on the outcomes. Moreover, it should also be noted that the encapsulation strategy achieves another important objective, namely, fat reduction, which cannot be obtained with the direct addition of oil.

Conversely, the reformulation favored a significant (*p* < 0.001) increase in protein in comparison with the CON group. This behavior could be related to the fact that the substitution of the animal fat by vegetable and marine oils corresponds to increments in the lean ratio and, as a consequence, in the protein proportion. Ash contents were also significantly (*p* < 0.001) higher in the reformulated samples than in the CON ones. As can be seen in Table 1, in this case, the composition of the fat source used seems to be reflected in the final product. Actually, the foal fat was composed of only 4.26% of protein and 0.13% of ash, while the microencapsulated oil mixtures contained higher (*p* < 0.001) amounts— 28.12% (T1) and 30.21% (T2) of protein and 1.37% (T1) and 1.47% (T1) of ash—probably due to the microcapsule wall material composition (sodium caseinate and lactose). These findings are in agreement with those of Vargas-Ramella et al. [21], who encapsulated other healthy oils via the same procedure as animal fat replacers. On the contrary, it was observed that the direct application of fresh fish oil [27], the use of ethyl cellulose and beeswax oleogels with a mixture of olive, linseed and fish oils [7], or beeswax oleogel with linseed oil [4] caused a decrease in protein percentages in the same type of product. This fact confirmed that the type and, especially, the composition of the structure selected for the oil incorporation can affect the composition of the final product.

Furthermore, the partial replacement of animal fat by the T1 and T2 microencapsulated oils had a significant (*p* < 0.001) effect on the instrumental color parameters (Table 3). In particular, a decrease in lightness in the experimental batches was observed, with the lowest values being reported for the T1 group (*p* < 0.01) followed by the T2 group, although, in this case, without significant differences (*p* > 0.05) in comparison with the control samples. According to studies in the literature [4,42], this behavior could be related to the diminution of the animal fat portions in the reformulated batches, since this fat is generally white and contributes a brilliant aspect to meat products [42]. Actually, it is recognized that fat amounts highly affect L* values, and different studies have observed that, as fat portions were increased, the lightness of products also increased [4,43]. Moreover, our values for the CON batch are close to the values published for foal pâté [44] (53–54%), while the outcomes obtained from the reformulated samples were in agreement with the range of values proposed by Vargas-Ramella et al. [21] (51–53%) in their study of microencapsulated oils as potential pork fat substitutes. At the same time, reformulation favored a significant (*p* < 0.001) increase in red hues in the T1 and T2 batches, while the control samples showed the lowest values. This tendency was also described by other researchers, who incorporated fish [27], tiger nut [21] and canola [45] oils in pâté using different forms. However, one of the above-cited authors [21] observed that the use of chia and linseed oils incorporated as microencapsulated powders did not alter the a* values in reformulated deer pâté. Considering b* values, also, this parameter showed increased (*p* < 0.001) values in the reformulated batches, with the highest levels of yellowness recorded for the T2 samples, while the lowest were recorded for the CON batches. These differences confirmed the results observed in previous studies on healthy pâté [4,21,22,27,45]. This behavior could be associated with the incorporation of oils, which contribute their generally yellow tonality. Thus, it could be expected that the color of this product would change and take on a more yellowish tone [4]. However, considering our findings, and in line with other authors [21], factors such as the type of oil chosen for the reformulation as well as the typical colors of the emulsifiers or oleogelators used, the quantity of oil employed and the ingredients included in the recipe for the elaboration of a meat product could account for the distinct results in the literature. 

Conversely, a consistent effect of the reformulation on pH values was not observed (*p* > 0.05), with results being similar among the three treatments. Additionally, all batches presented values consistent with those found for this type of meat product (5.86–6.07) [5,21]. According to our outcomes, other researchers [21,22,27] also observed that the partial substitution of animal fat by healthy oils used in different forms in pâté did not affect the pH values of samples. 

As regards lipid stability, traditional pâté is a highly processed product characterized by elevated amounts of fat and low contents of natural antioxidants, and, as a consequence, it is generally defined as a type of meat product particularly vulnerable to oxidation [40,46]. Furthermore, the manufacturing process is the phase in which different factors could cause an increase in oxidative reactions: diminution in particle size during the preparation facilitates the exposure of unstable compounds to oxygen; salting could favor the pro-oxidant activity of iron in myofibrillar foods; and thermal treatment generally provokes protein denaturation, decline in enzymatic activity and the release of non-haem iron (catalyzing lipid oxidation) [40]. In addition, as was previously pointed out, lipid oxidation represents one of the main technological issues to be faced when the lipid fraction of a meat product has a higher content of unsaturated fatty acids after non-meat fats have been incorporated, such as the oils employed in our case (Table 2) [40,47]. Nonetheless, although these conditions were present in this study, the T1 and T2 batch results were unaffected (*p* > 0.05), with lipid oxidation showing similar values to the CON samples (0.63 mg MDA/kg sample for the CON and T1 groups, 0.59 mg MDA/kg sample for the T2 group). Moreover, it is important to underline that our outcomes remained below the sensory threshold limit at which consumers perceive rancidity (2.0 mg MDA/kg sample) [42,48]. A similar trend was observed in a previous investigation [21], where microencapsulated tiger nut oil was employed as a partial fat replacer in deer pâtés. Contrarily, the same authors noted that the incorporation of microencapsulated chia or linseed oils increased the lipid oxidation of the samples. This same tendency was observed, also, by other researchers [27,40]. However, in the case of healthier reformulations, these discrepancies could be attributed to the amount, type and method of incorporation of the lipid source, the type of product and raw materials employed, as well as the potential presence and action of natural antioxidant compounds [21,40,49]. Our promising results, in fact, could be related to the protective action of the microcapsules against the oxidizing agents [50] and the presence of natural antioxidants in the oils employed [18,49,51]. This fact could be confirmed by data shown in Table 1, the microencapsulated oil mixtures presenting TBAR values lower (*p* < 0.05) than foal fat. It is important to note that canned pâtés are characterized by the absence of oxygen, which is a necessary molecule for oxidative processes [10,52,53]. Therefore, this anoxia caused oxidative reactions to be controlled, such that there were no differences between the batches. Finally, some authors [40] have suggested that the high temperature obtained at the thermal center of the pâté (about 80 °C) could explain our outcomes. Concretely, Maillard reaction products are generally formed starting from this temperature and can act as oxidation protectors [40].

The textures of our samples followed the same behavior observed for pH and TBAR parameters. Indeed, also in this case, the partial replacement of the animal fat by T1 and T2 microcapsules did not alter (*p* > 0.05) the penetration force values, which were similar values for all treatments (0.48, 0.53 and 0.51 N for the CON, T1 and T2 groups, respectively). Our outcomes contrast with those of another study in the literature [21], in which it was reported that the fat amounts and characteristics generally highly affected the rheological properties of meat products. Moreover, the presence of high levels of unsaturated fatty acids in oils generally confers softer textures to samples in which animal fats are totally or partially replaced by healthy oils [21,22,27,54]. In our case, as can be seen in Table 1 and Table 2, the horse fat and the microencapsulated oils presented completely different fat percentages and compositions, as was mentioned earlier. Moreover, it is evident that the fat sources employed in this study had distinct consistencies: the microencapsulated oil mixtures are dry powders obviously characterized by different texture features in comparison with animal fat. However, despite the compositional and rheological dissimilarities, our data indicate that these differences disappeared once the microencapsulated oil mixtures were incorporated and were submitted to the thermal and mechanical processes necessary for the elaboration of the product [7]. Hence, our outcomes are encouraging and suggest that, probably, the microcapsule formulations were able to reproduce the meat-binding properties that the animal fat presented in the CON pâtés. Furthermore, our samples showed values lower than those found by other authors [7,21] and were characterized by a soft consistency (subjective assessment). On the whole, our findings and the discrepancies found in comparing them with others in the literature are difficult to attribute to a unique cause, since several factors could be involved, such as the type and quantity of raw materials employed, the selected oils and their incorporation forms, the interaction of the animal fat replacers with meat and the processing protocols for the meat product elaboration, among others. However, it is worth highlighting that the use of these healthier lipid sources as partial animal fat replacers showed their suitability with regard to texture, which remained unaffected.

### 3.2. Fatty Acid Composition and Health Indices of the Foal Pâtés

Table 4 shows the impact of the use of T1 and T2 as partial animal fat replacers on the fatty acid profiles (g/100 g of fat) of the foal pâtés (only those represented ≥0.1%). As can be seen, the fat reformulation significantly (*p* < 0.001) affected the lipid profiles of the final products, except for three singular fatty acids (elaidic-11t-C18:1; *cis*-9, *trans*-11-linolenic-9c,11t-C18:2, and docosapentaenoic-C22:5*n*-3). In detail, monounsaturated fatty acids (MUFAs) represented the most abundant group in the CON and T2 batches (36.02 and 38.86 g/100 g of fat, respectively), followed, in decreasing order, by SFAs (34.13 and 30.52 g/100 g of fat, respectively) and PUFAs (18.57 and 22.04 g/100 g of fat, respectively) (MUFAs > SFAs > PUFAs), while, in the T1 samples, PUFAs (31.18 g/100 g of fat) were the most plentiful group, followed by similar contents of MUFAs (31.13 g/100 g of fat), and SFAs (28.89 g/100 g of fat) were the minority fraction (PUFAs ≥ MUFAs > SFAs). Moreover, taking into consideration fatty acids individually, oleic acid (C18:1*n*-9) represented the most abundant fatty acid in all the batches. In the case of the CON and T2 pâtés, palmitic acid (C16:0) followed by linoleic (C18:2*n*-6) and α-linolenic (C18:3*n*-3) acids were other majority fatty acids, taking the following order C18:1*n*-9 > C16:0 > C18:2*n*-6 > C18:3*n*-3, while, in the T1 group, C18:2*n*-6, C16:0 and C18:3*n*-3, in decreasing order, represented the second, third and fourth most plentiful fatty acids (C18:1*n*-9 > C18:2*n*-6 > C16:0 > C18:3*n*-3).

Concerning SFAs, fat reformulation favored a remarkable (*p* < 0.001) decrease in their amounts. Actually, the T1 and T2 samples showed reductions of 15.35 and 10.57%, respectively, in comparison with the CON group, principally related to the significant (*p* < 0.001) decrease in miristic (C14:0), palmitic (C16:0) and stearic (C18:0) fatty acids. This finding, as previously commented, could be expected, since the animal fat, known for its high SFA contents, was partially replaced by healthy oils (walnut or pistachio oil mixed with algal oil), presenting lower (*p* < 0.001) amounts of this group of fatty acids (especially C16:0 and C18:0) (Table 2). Furthermore, as previously pointed out, and consistent with previous research [31,32,55], the decrease in SFA concentrations also promoted reductions in the atherogenic, hypercholesterolaemic and thrombogenic (C14:0, C16:0 and C18:0) effects, since some of the fatty acids in this group can have different impacts on the cholesterol concentrations in distinct plasma lipoprotein fractions, as was earlier pointed out [32]. 

As regards MUFAs, significant differences (*p* < 0.001) were detected among batches, with the highest values reported for the T2 samples, followed by the CON and T1 groups. These results could be related to the significant (*p* < 0.001) increase in C18:1*n*-9 values in the T2 pâtés, probably due to the fact that this fatty acid is predominant in pistachio oil (Table 2). A similar tendency was also reported in other studies, where pâtés were reformulated using microencapsulated tiger nut oil [21] and oleogels containing mixtures of olive, linseed and fish oils [7] or olive oil [22] as animal fat substitutes. 

PUFA contents, also, were significantly (*p* < 0.001) affected by the type of fat source employed. In particular, the use of microencapsulated oil mixtures favored a relevant (*p* < 0.001) increment in PUFA contents; in this case, T1 samples showed the highest concentrations, followed by the T2 and CON samples. Considering PUFAs individually, C18:2*n*-6, which was predominant in all treatments, increased significantly (*p* < 0.001) in both reformulated batches, although the highest values were reported for T1. This outcome could also explain the highest (*p* < 0.001) *n*-6 values shown in this batch compared with the other two groups of samples. In addition, considering *n*-3 fatty acids, only the batch reformulated using walnut oil mixed with algal oil (T1) showed greater (*p* < 0.001) values. C18:3*n*-3 was the most plentiful *n*-3 fatty acid in all batches, although the T1 pâtés contained the highest (*p* < 0.001) contents. Our outcomes seem to reproduce the lipid profile of walnut oil, both of these single fatty acids (C18:2*n*-6 and C18:3*n*-3) representing the most abundant PUFAs (Table 2). These results are in line with those obtained by Botella-Martinez et al. [56], who also studied the incorporation of this oil as a fat replacer in beef burgers. Our findings are in agreement with those obtained in recent investigations [7,9,21,27], where PUFA contents were significantly increased in pâtés elaborated using vegetable and/or marine oils as partial or total animal fat replacers, and *n*-6 and/or *n*-3 increased based on the selected oils. Moreover, it is worth highlighting that our CON samples showed higher C18:3*n*-3 contents than those detected in the same product elaborated using pork back fat as a lipid source [21,22,44]. In fact, as commented, equids are characterized by the capability to absorb and transfer dietary *n*-3 PUFAs, such as C18:3*n*-3, from feed to muscle tissue [20,57]. A favorable deposition of this fatty acid was observed both in the fat (Table 2) and in the meat [58] employed in this study in comparison with data for pork [44,59,60], which is the meat source most commonly employed in the elaboration of pâtés. Thus, as could be expected, the “beneficial” lipid profile, which generally distinguishes foals, was also reflected in our final product and represented a promising starting point to obtain healthy meat products. Furthermore, the incorporation of healthy oils certainly could further improve the nutritional quality of the final product. In addition, another interesting aspect is that the use of algal oil for the formulation of the oil mixtures also contributed to the significant increase in *n*-3 values in the T1 samples and in long-chain *n*-3 (LC *n*-3) values in both of the reformulated pâtés. In fact, the lipid profile of this marine oil, characterized by elevated levels of LC *n*-3, as EPA and DHA [14], is also reflected in our outcomes. Values of 201.02 and 182.33 mg EPA + DHA/100 g of product were recorded for the T1 and T2 groups, respectively. European Regulation No. 116/2010 [39] requires that a product contains a minimum of 40 and 80 mg of the sum of EPA + DHA/100 g of product to be claimed a “source of omega-3 fatty acids” and to have a “high omega-3 content”. In this regard, these claims can be made for both of the reformulated batches. Hence, this is another positive aspect of our lipid reformulations. As is well-known, foods rich in *n*-3 PUFAs (such as EPA and DHA) can lower the incidence of cardiovascular diseases (CVDs) and play a protective role against inflammatory and neurodegenerative illnesses [17,32].

On the whole, our findings are in agreement with those of other studies in the scientific literature [21,61], where it was observed that the use of microencapsulated healthy oils as animal fat replacers produced relevant reductions in SFA amounts and significant increases in unsaturated fatty acids, MUFAs and/or PUFAs. Henceforth, in light of our results, the fatty acid profiles of our products seem to be in line with the lipid compositions and amounts of the fat sources employed. The same conclusion was obtained by different authors [16,21,62] studying healthy lipid reformulations in meat products. Nevertheless, the discrepancies present in the literature could be associated with various factors, not only the type and quantity of oils used in formulations but also the forms in which the oils are incorporated, the raw materials chosen, the amounts of animal fat replaced (partial or total) and the manufacturing processes, among others. 

In conclusion, considering the nutritional properties of foal pâté, as could be expected, the changes observed in the *n*-6 and *n*-3 parameters affected the *n*-6/*n*-3 ratio, which showed significant (*p* < 0.001) increases in the T1 and T2 samples. Nevertheless, the results obtained for all batches were below 4, respecting the nutritional recommendations for human diets proposed by the Food and Agriculture Organization (FAO) [63]. Vargas-Ramella et al. [21] also observed an increase in this ratio in deer pâté reformulated with microencapsulated tiger nut oil, although, in this case, the values obtained were much higher than ours and did not respect the health limit suggested. Nevertheless, this health ratio cannot be considered singularly. The fat reformulations favored increments (*p* < 0.001) in PUFA/SFA ratios, with values greater than 0.45 recorded (1.08 and 0.73 for the T1 and T2 samples, respectively), as suggested by other studies [64]. Similarly, other authors [7,9,56] observed this same trend. Furthermore, AI and TI indices, as previously discussed, reveal potential levels of platelet aggregation. Thus, the lower the AI and TI values, the healthier could be the final product, since there is greater potential for the lipid portion to prevent the occurrence of CVDs [30,32], while, concerning the h/H index, these values should be high in order to achieve greater hypocholesterolaemic effects. In this context, our outcomes met these health indications, too: the partial replacement of foal fat in the T1 and T2 samples significantly reduced (*p* < 0.001) the AI and TI indices and increased (*p* < 0.001) the h/H ratio in comparison with the traditional product (CON). Thus, it can be concluded that these microencapsulated oil mixtures improved the fatty acid profiles of our final products and that the T1 batch represented an encouraging formulation from a nutritional standpoint. Moreover, algal oil stood out for its action in the lipid profiles of the samples. Actually, despite high SFA and low MUFA amounts, it favored a remarkable increase in LC *n*-3 (valuable healthy fatty acids), as previously commented. Additionally, another factor to underline is that, although the quantity of algal oil used for the oil mixture was minimal (7 g/100 g of oil mixture), in order to avoid negative effects on the sensory attributes of the final product, it was sufficient to promote the improvement of the T1 and T2 batches so as to warrant certain health claims. Hence, it is evident that the mixture of this “precious” oil with other healthy oils and its inclusion in the form of microcapsules in foal pâté demonstrated a promising strategy and a solution to obtain products with enhanced lipid profiles and nutritional properties.

### 3.3. Sensory Analysis of the Foal Pâtés

The results obtained from the acceptance test are shown in Table 5. Statistical analysis showed that no significant (*p* > 0.05) differences were found in any of the attributes evaluated in our samples, indicating that the employment of the T1 and T2 animal fat replacers did not alter the acceptability of the pâtés. Actually, consumers rated each descriptor with similar scores for the three groups of samples. Only in the case of overall acceptability was a different tendency observed (*p* < 0.1), with T2 samples being assigned values similar to the CON ones, while the T1 group received the lowest scores. In this context, the texture data corresponded with those obtained from the instrumental analysis, while the color differences detected instrumentally seemed to not influence the evaluation of the consumers regarding the visual aspect (Table 3). In addition, all formulations were “accepted”, since the obtained values were higher than 4, which was the acceptability limit. Thus, our outcomes can be considered encouraging. In comparison with other studies, Vargas-Ramella et al. [21], who also employed microencapsulation for oil incorporation in pâté, found that the acceptability results were unaltered when tiger nut oil was used, while in the case of linseed and chia oils, the values decreased significantly in comparison to the control. Furthermore, our samples, including the T2 microcapsules, presented higher global acceptability than those obtained for the reformulated batches by the above-mentioned authors. In the same way, the results obtained for the T1 samples seemed to be better or more optimistic than those published by other authors studying the application of walnut oil in meat products [56,65].

As regards the preference test, it can help to understand how consumer perceptions change when they have to choose one treatment considering any singular attribute. In this case, according to the total scores of preferences (number in brackets in Table 6), the T1 samples were preferred for visual aspect and odor, while the CON samples were preferred for taste and T2 for texture, although without significant differences (F_test_ < F = 0.05). Thus, consumers indicated similar preferences for the traditional (CON) and reformulated products considering these attributes, while they chose first, at the same level, the greasy character of the CON and T2 samples, with the T1 group the least favorite in comparison with the control (F_test_ > F = 0.05). Thus, this aspect could be at the base of the results obtained for the global preference, with T2 and CON being the most preferred, while the T1 samples were the least, with significant differences (F_test_ > F = 0.05). This result could be related to the fact that the samples elaborated using microencapsulated walnut and algal oils also had the lowest fat contents (Table 3); it is possible that consumers perceived this difference.

Nevertheless, on the basis of our results for the hedonic analysis, it can be concluded that sensory acceptability did not change when T1 and T2 microcapsules were used as partial animal fat replacers, and all samples were shown to be accepted as satisfactory, although differences were observed in terms of preference.

## 4. Conclusions

The obtained results showed that the use of microcapsules containing blends of algal oil and walnut (T1) or pistachio (T2) oils as partial animal fat replacers favored the diminution of fat contents and the enhancement of the nutritional profiles of foal pâtés, warranting the claims of “reduced fat content” (T1), being a “source of omega-3 fatty acids” and of having a “high omega-3 content” (T1 and T2). Moreover, despite the changes observed, the texture and lipid oxidation of the samples were unaltered by the fat reformulation. Nevertheless, sensory acceptability was unaffected by the incorporation of T1 and T2 microcapsules. On the other hand, further investigations are required to improve technological aspects of the reformulated samples, such as the color.

In addition, the employment of equid raw materials (subcutaneous fat, lean meat and liver) demonstrated a new starting point for the meat industry to obtain a ready-to-eat product with added value, not only from a nutritional standpoint but also from an environmental point of view, in line with modern market requirements.

Hence, as a general conclusion, our findings suggest that the incorporation of these microencapsulated oil mixtures as partial animal fat replacers in this type of meat product, the T1 treatment in particular, could represent a successful strategy to obtain healthy foal pâtés, without compromising consumer acceptance.

## Figures and Tables

**Table 1:** Chemical composition and lipid oxidation of foal dorsal fat and microencapsulated oils.

Parameters	Horse Fat	Microencapsulated Oils		SEM	*p*-Value
		Walnut and Algal Oils	Pistacho and Algal Oils		
Moisture (g/100 g)	10.25 ^c^	0.43 ^a^	1.18 ^b^	1.084	0.001
Fat (g/100 g)	85.56 ^b^	21.54 ^a^	22.84 ^a^	7.252	0.001
Protein (g/100 g)	4.26 ^a^	28.12 ^b^	30.21 ^c^	2.856	0.001
Ash (g/100 g)	0.13 ^a^	1.37 ^b^	1.47 ^c^	0.148	0.001
TBARS (mg MDA/kg)	0.57 ^b^	0.46 ^a^	0.46 ^a^	0.020	0.016

^a–c^ Mean values in the same row (corresponding to the same parameter) with different letters differ significantly (*p* < 0.05; Duncan’s test); SEM: Standard error of the mean.

**Table 2:** Fatty acid composition (expressed as g/100 g of fat) of foal subcutaneous fat and oils employed for pâté elaboration.

Fatty Acids	Fat Source				SEM	*p*-Value
	Horse Fat	Walnut Oil	Pistacho Oil	Seaweed Oil		
C10:0	0.11 ^c^	0.05 ^b^	0.05 ^b^	0.02 ^a^	0.010	0.001
C12:0	0.25 ^b^	0.04 ^a^	0.04 ^a^	0.61 ^c^	0.056	0.001
C14:0	4.09 ^b^	0.05 ^a^	0.11 ^a^	7.74 ^c^	0.774	0.001
C14:1*n*-5	0.39 ^c^	0.00 ^a^	0.00 ^a^	0.11 ^b^	0.048	0.001
C15:0	0.34 ^b^	0.03 ^a^	0.02 ^a^	0.51 ^c^	0.052	0.001
C16:0	27.63 ^d^	5.94 ^a^	10.33 ^b^	15.25 ^c^	2.381	0.001
C16:1*n*-7	6.99 ^d^	0.09 ^a^	0.96 ^b^	5.95 ^c^	0.814	0.001
C17:0	0.37 ^c^	0.06 ^a^	0.06 ^a^	0.17 ^b^	0.037	0.001
C18:0	3.32 ^d^	2.26 ^c^	1.28 ^b^	0.49 ^a^	0.309	0.001
9*t*-C18:1	0.11 ^d^	0.02 ^a^	0.07 ^c^	0.04 ^b^	0.010	0.001
11*t*-C18:1	0.27 ^b^	0.26 ^b^	0.24 ^b^	0.00 ^a^	0.028	0.001
C18:1*n*-9	32.30 ^c^	17.35 ^b^	49.17 ^d^	0.11 ^a^	4.410	0.001
C18:1*n*-7	1.60 ^b^	0.81 ^a^	2.14 ^c^	5.50 ^d^	0.440	0.001
C18:2*n*-6	11.90 ^b^	54.86 ^d^	27.27 ^c^	0.06 ^a^	5.062	0.001
C18:3*n*-3	4.37 ^b^	11.84 ^c^	0.46 ^a^	0.01 ^a^	1.141	0.001
9*c*,11*t*-C18:2 (CLA)	0.28 ^c^	0.22 ^b^	0.22 ^b^	0.00 ^a^	0.029	0.001
C20:0	0.08 ^b^	0.12 ^c^	0.17 ^d^	0.02 ^a^	0.013	0.001
C20:1*n*-9	0.47 ^d^	0.18 ^b^	0.31 ^c^	0.00 ^a^	0.049	0.001
C20:2*n*-6	0.27 ^c^	0.04 ^b^	0.03 ^b^	0.00 ^a^	0.033	0.001
C20:4*n*-6	0.09 ^b^	0.00 ^a^	0.00 ^a^	0.20 ^c^	0.020	0.001
C20:3*n*-3	0.17 ^b^	0.00 ^a^	0.00 ^a^	0.00 ^a^	0.022	0.001
C22:0	0.04 ^b^	0.06 ^c^	0.12 ^d^	0.00 ^a^	0.011	0.001
C20:5*n*-3 (EPA)	0.04 ^a^	0.00 ^a^	0.00 ^a^	1.33 ^b^	0.142	0.001
C22:5*n*-3 (DPA)	0.23 ^c^	0.00 ^a^	0.00 ^a^	0.16 ^b^	0.028	0.001
C22:6*n*-3 (DHA)	0.04 ^a^	0.00 ^a^	0.07 ^a^	41.83 ^b^	4.476	0.001
SFA	36.39 ^d^	8.80 ^a^	12.42 ^b^	24.88 ^c^	3.108	0.001
MUFA	42.12 ^c^	18.79 ^b^	52.97 ^d^	11.71 ^a^	4.174	0.001
PUFA	17.50 ^a^	66.96 ^d^	28.07 ^b^	43.75 ^c^	5.033	0.001
*n*-3	4.85 ^b^	11.84 ^c^	0.53 ^a^	43.33 ^d^	4.168	0.001
*n*-6	12.35 ^b^	54.90 ^d^	27.31 ^c^	0.42 ^a^	5.022	0.001
LC *n*-3	0.31 ^a^	0.00 ^a^	0.07 ^a^	43.33 ^b^	4.622	0.001

^a–d^ Mean values in the same row (corresponding to the same parameter) with different letters differ significantly (*p* < 0.05; Duncan’s test). SEM: Standard error of the mean; SFA: Saturated fatty acid; MUFA: Monounsaturated fatty acid; PUFA: Polyunsaturated fatty acid; *n*-3: Omega-3; *n*-6: Omega-6; LC *n*-3: Long-chain omega-3.

**Table 3:** Proximate composition, lipid oxidation and physicochemical properties of foal pâtés.

Parameters	Treatments			SEM	*p*-Value
	CON	T1	T2		
*Chemical composition*					
Moisture (g/100 g)	52.22 ^c^	50.81 ^b^	49.71 ^a^	0.162	0.001
Fat (g/100 g)	26.97 ^c^	17.74 ^a^	19.37 ^b^	0.538	0.001
Protein (g/100 g)	15.46 ^a^	19.14 ^b^	19.34 ^b^	0.235	0.001
Ash (g/100 g)	3.24 ^a^	3.43 ^b^	3.42 ^b^	0.013	0.001
*Physicochemical parameters*					
L*	53.08 ^b^	51.74 ^a^	52.73 ^b^	0.167	0.002
a*	11.35 ^a^	13.15 ^c^	12.60 ^b^	0.131	0.001
b*	20.20 ^a^	21.46 ^b^	22.35 ^c^	0.169	0.001
pH	6.04	6.03	6.06	0.012	0.682
TBARS (mg MDA/kg)	0.63	0.63	0.59	0.017	0.437
*Penetration test*					
Penetration force (N)	0.48	0.53	0.51	0.010	0.171

^a–c^ Mean values in the same row (corresponding to the same parameter) with different letters differ significantly (*p* < 0.05; Duncan’s test). SEM: Standard error of the mean. CON: pâté prepared with 100% foal fat; T1: pâté prepared with 50% of foal fat replaced by microencapsulated walnut and algal oil mixture; T2: pâté prepared with 50% of foal fat replaced by microencapsulated pistachio and algal oil mixture.

**Table 4:** Fatty acid compositions (expressed as g/100 g of fat) of the foal pâtés.

Fatty Acids	Treatments			SEM	*p*-Value
	CON	T1	T2		
C8:0	0.09 ^a^	0.10 ^b^	0.10 ^b^	0.001	0.001
C10:0	0.15 ^a^	0.16 ^b^	0.16 ^b^	0.001	0.001
C12:0	0.28 ^a^	0.29 ^a^	0.31 ^b^	0.002	0.001
C14:0	3.30 ^c^	2.73 ^a^	3.07 ^b^	0.052	0.001
C14:1*n*-5	0.27 ^b^	0.22 ^a^	0.27 ^b^	0.006	0.001
C15:0	0.28 ^b^	0.25 ^a^	0.28 ^b^	0.003	0.001
C16:0	24.89 ^c^	20.47 ^a^	22.10 ^b^	0.275	0.001
C16:1*n*-7	5.12 ^b^	4.08 ^a^	4.93 ^b^	0.089	0.001
C17:0	0.36 ^b^	0.28 ^a^	0.29 ^a^	0.004	0.001
C18:0	4.58 ^c^	4.34 ^b^	3.91 ^a^	0.046	0.001
9*t*-C18:1	0.12 ^c^	0.10 ^a^	0.11 ^b^	0.001	0.001
11*t*-C18:1	0.28	0.29	0.29	0.003	0.345
C18:1*n*-9	28.41 ^b^	24.70 ^a^	31.19 ^c^	0.355	0.001
C18:1*n*-7	1.40 ^a^	1.37 ^a^	1.67 ^b^	0.022	0.001
C18:2*n*-6	11.20 ^a^	21.06 ^c^	14.68 ^b^	0.545	0.001
C18:3*n*-3	5.87 ^b^	7.85 ^c^	5.17 ^a^	0.170	0.001
9*c*,11*t*-C18:2 (CLA)	0.28	0.29	0.29	0.002	0.351
C20:0	0.08 ^a^	0.09 ^b^	0.10 ^c^	0.001	0.001
C20:1*n*-9	0.42 ^c^	0.34 ^a^	0.36 ^b^	0.006	0.001
C20:2*n*-6	0.25 ^b^	0.20 ^a^	0.20 ^a^	0.003	0.001
C20:4*n*-6	0.25 ^a^	0.29 ^b^	0.30 ^b^	0.004	0.001
C20:3*n*-3	0.20 ^b^	0.18 ^a^	0.18 ^a^	0.002	0.001
C20:5*n*-3 (EPA)	0.07 ^a^	0.10 ^b^	0.10 ^b^	0.002	0.001
C22:5*n*-3 (DPA)	0.24	0.24	0.25	0.002	0.464
C22:6*n*-3 (DHA)	0.06 ^a^	0.82 ^c^	0.73 ^b^	0.044	0.001
SFA	34.13 ^c^	28.89 ^a^	30.52 ^b^	0.337	0.001
MUFA	36.02 ^b^	31.13 ^a^	38.86 ^c^	0.429	0.001
PUFA	18.57 ^a^	31.18 ^c^	22.04 ^b^	0.722	0.001
*n*-3	6.44 ^a^	9.20 ^b^	6.43 ^a^	0.190	0.001
*n*-6	11.82 ^a^	21.67 ^c^	15.29 ^b^	0.545	0.001
LC *n*-3	0.37 ^a^	1.17 ^c^	1.08 ^b^	0.047	0.001
*n*-6/*n*-3	1.84 ^a^	2.37 ^b^	2.38 ^b^	0.034	0.001
PUFA/SFA	0.55 ^a^	1.08 ^c^	0.73 ^b^	0.031	0.001
TI	0.75 ^c^	0.51 ^a^	0.62 ^b^	0.015	0.001
AI	0.70 ^c^	0.51 ^a^	0.57 ^b^	0.012	0.001
h/H	1.68 ^a^	2.39 ^c^	2.11 ^b^	0.043	0.001

^a–c^ Mean values in the same row (corresponding to the same parameter) with different letters differ significantly (*p* < 0.05; Duncan’s test). SEM: Standard error of the mean. CON: pâté prepared with 100% foal fat; T1: pâté prepared with 50% of foal fat replaced by microencapsulated walnut and algal oil mixture; T2: pâté prepared with 50% of foal fat replaced by microencapsulated pistachio and algal oil mixture. SFA: Saturated fatty acids; MUFA: Monounsaturated fatty acids; PUFA: Polyunsaturated fatty acids; *n*-3: Omega-3; *n*-6: Omega-6; LC *n*-3: Long-chain omega-3; TI: Thrombogenic index; AI: Atherogenic index; h/H: Hypo/hypercholesterolemic fatty acid ratio.

**Table 5:** Acceptance test results for the foal pâtés.

Attributes	Treatments			SEM	*p*-Value
	CON	T1	T2		
Visual aspect	5.7	5.9	6.1	0.088	0.266
Odor	5.3	5.4	5.5	0.096	0.681
Taste	5.4	5.3	5.5	0.106	0.627
Texture	5.2	5.2	5.3	0.116	0.941
Greasy character	5.5	5.2	5.2	0.103	0.446
Overall acceptability	5.5	4.9	5.3	0.101	0.072

SEM: Standard error of the mean. CON: pâté prepared with 100% foal fat; T1: pâté prepared with 50% of foal fat replaced by microencapsulated walnut and algal oil mixture; T2: pâté prepared with 50% of foal fat replaced by microencapsulated pistachio and algal oil mixture.

**Table 6:** Preference test results for the foal pâtés.

	Sample Most Favored		Sample Least Favored
Visual aspect	T1 (43)	T2 (40)	CON (37)
		
	F_test_ = 2.70 < F(α = 0.05) = 5.99		
Odor	T1 (41)	CON (41)	T2 (38)
		
	F_test_ = 0.90 < F(α = 0.05) = 5.99		
Taste	CON (42)	T1 (40)	T2 (38)
		
	F_test_ = 1.20 < F(α = 0.05) = 5.99		
Texture	T2 (42)	CON (40)	T1 (38)
		
	F_test_ = 1.20 < F(α = 0.05) = 5.99		
Greasy character	CON (138)	T2 (117)	
		T2 (117)	T1 (105)
		
	F_test_ = 9.30 > F(α = 0.05) = 5.99		
Global preference	T2 (129)	CON (129)	
			T1 (102)
		
	F_test_ = 8.10 > F(α = 0.05) = 5.99		

Samples in the same row (orange) did not show significant differences (*p* > 0.05); samples in different rows showed significant differences (*p* < 0.05). Numbers in brackets are Σ scores. CON: pâté prepared with 100% foal fat; T1: pâté prepared with 50% of foal fat replaced by microencapsulated walnut and algal oil mixture; T2: pâté prepared with 50% of foal fat replaced by microencapsulated pistachio and algal oil mixture.

## Data Availability

All data are presented in the manuscript.
